# Endoscopic Septoturbinoplasty among Patients Undergoing Surgery in the Department of Otolaryngology-Head and Neck of a Tertiary Care Hospital: A Descriptive Cross-sectional Study

**DOI:** 10.31729/jnma.7212

**Published:** 2021-12-31

**Authors:** Nain Bahadur Mahato, Meera Bista, Bhuwan Bhandari, Rosi Pradhan

**Affiliations:** 1 Department of Otolaryngology-Head and Neck Surgery, Kathmandu Medical College and Teaching Hospital, Sinamangal, Kathmandu, Nepal; 2 Department of Surgery, Nepal National Hospital, Kalanki, Kathmandu, Nepal; 3 Department of Anaesthesia and Critical Care, KIST Medical College, Imadol, Lalitpur, Nepal

**Keywords:** *endoscopy*, *Nepal*, *septoplasty*, *surgery*, *turbinoplasty*

## Abstract

**Introduction:**

Septoplasty without turbinoplasty is one of the main reasons for failure of procedure in case of deviated nasal septum with inferior turbinate hypertrophy. Septoturbinoplasty is the procedure of choice for complete treatment. The main objective is to find out the prevalence of endoscopic septoturbinoplasty among patients undergoing surgery in Department of Otolaryngology-Head and Neck of a tertiary care hospital.

**Methods:**

This was a descriptive cross-sectional study in Department of Otolaryngology-Head and Neck Surgery done over a period of 1 year duration from 1st August 2020 to 31st July 2021 among 1248 patients who underwent surgery in the department. Ethical Approval was taken from Institutional Review Committee of Kathmandu Medical College and Teaching Hospital (Reference number: 2207202004). A convenience sampling technique was used. Two different techniques, Microdebrider Assisted Turbinoplasty and Out-fracture with Submucosal Diathermy were used in surgery. Nasal Obstruction and Symptom Evaluation Scale questionnaire was used pre and postoperatively for data collection. Data were analyzed in Statistical Package for the Social Sciences version 16. Point estimate at 95% Confidence Interval was calculated, with frequency, percentage, mean and standard deviations.

**Results:**

Out of 1248 patients, about 92 (7.37%) patients (4.95-10.14 at 95% Confidence Interval) underwent septoturbinoplasty. The mean Nasal Obstruction and Symptom Evaluation Scale before surgery was 75.21±6.19.

**Conclusions:**

The prevalence of septoturbinoplasty in our study is similar to other studies done in similar settings. We found almost complete resolution of breathing problems following endoscopic septoturbinoplasty, hence improving quality of life.

## INTRODUCTION

Nasal obstruction negatively affects patients' quality of life (QOL).^1-3^ Sustained nasal obstruction may be due to deviation of the nasal septum (DNS) associated with compensatory hypertrophy of inferior turbinate (ITH).^4^ Septoturbinoplasty is a corrective surgical procedure done to straighten a deviated nasal septum along with reduction in size of turbinates. The most common cause of septoplasty failure is inferior turbinate hypertrophy (ITH) that is not treated properly.^5^

Several techniques have been described for the correction of inferior turbinate hypertrophy such as total or partial turbinectomy, submucosal diathermy, microdebrider assisted turbinoplasty, out fracture, radiofrequency, laser therapy etc.^6-9^ Despite the increasing number of surgical procedures turbinoplasty, out fracture, and bipolar cautery methods are frequently in use for the last three decades.^10^

The main objective is to find out the prevalence of endoscopic septoturbinoplasty among patients undergoing surgery in Department of Otolaryngology-Head and Neck of a tertiary care center.

## METHODS

This was a descriptive cross-sectional study in the department of Otolaryngology-Head and Neck Surgery (HNS) of Kathmandu Medical College and Teaching Hospital (KMCTH) over a period of one year duration from 1st August 2020 to 31st July 2021 AD. Ethical Approval was taken from the Institutional Review Committee of KMCTH (Reference number: 2207202004). All the patients who gave consent for the surgery were included in our study and who did not give the consent were excluded from the study. Patients having symptomatic deviated nasal septum with compensatory hypertrophy of inferior turbinates seeking septoturbinoplasty were included in our study. Patients with septal perforations, nasal polyposis, acute or chronic rhinosinusitis, concha bullosa, previous nasal or paranasal surgery and granulomatous diseases such as tuberculosis, syphilis, Wegener's granulomatosis were excluded from the study, also the patients who did not give the consent were excluded. A convenience sampling technique was used.

Sample size was calculated using the formula:

n = Z^2^ × p × q / e^2^

  = (1.96)^2^ × 0.5 × 0.5 / (0.03)^2^

  = 1068

Where,

n= minimum required sample size
Z= 1.96 at 95% Confidence Interval (CI)
p= 50% prevalence taken for maximum sample size calculation
q= 1-p
e= margin of error, 3%

Hence, the calculated sample size was 1068. However, we included 1248 patients.

Adding 10% missing data rate, the calculated sample size was 1175. However, 1248 samples were taken.

All the patients to be enrolled in the study group were examined by a consultant Ear, Nose, and Throat (ENT) surgeon. History regarding the cause of nasal obstruction either congenital or traumatic was taken. Diagnostic Nasal Endoscopy (DNE) using Fiberoptic Laryngoscopy (FOL) was done to find out the type and grade of deviated nasal septum (DNS) and to rule out any other endonasal pathology other than ITH. NOSE scale^11^ questionnaire comprising 5 questions and each question having five options between 0 and 4 was used for data collection. For all 5 questions the total score was calculated and the result was divided by 20 and multiplied by 100 (Zero represents no problem and 100 severe problems). The questions were asked to only those patients who had undergone septoturbinoplasty. The questionnaire was converted into both Nepalese and English version. The questions were asked as an interview so as to give maximum comfort to the patients. The patients were taught how to score the questionnaire in the postoperative period for data collection.

Two different techniques, Microdebrider Assisted Turbinoplasty and Out-fracture with Submucosal Diathermy were used. For the surgical procedure, informed written consent was taken from the patient and patient parties. The procedure was done under general anesthesia. Septoplasty with quilting suture on the septum was done in all the cases followed by reduction of ITH.

Microdebrider Assisted Turbinoplasty was performed to reduce the size of bulky inferior turbinate intraturbinally under endoscopic guidance. A submucosal pocket was dissected by tunneling with freer's elevator and microdebrider blade with dissecting tip in an anterior to posterior and superior to inferior sweeping motion. A 2.9mm diameter microdebrider tip (Medtronic Xomed®), rotating continuously in a circular fashion was set at 3,000rpm while using suction irrigation, was applied to remove all the stromal tissue from inside of the turbinate with preservation of the mucosal flap. For the reduction of hypertrophied posterior end (tails), a second entry point was made at the mid-portion of the inferior turbinate to gain better access to treat the ''mulberry-tip'' of the inferior turbinate.

Similarly, Out-fracture and Submucosal Diathermy techniques were also used. Under endoscopic guidance, out-fracture of inferior turbinate was done by the use of Freer's elevator on both sides and then a 3-4mm mucosal incision was made on the head of the inferior turbinate. The tunnel was created on the medial surface and inferior edge of the bone by the use of freer's elevator. The excess submucosal and cavernous tissue was cauterized by using bipolar electrocautery forceps set at the power of 35-45W in the posterior to anterior direction.

After completion of the surgery, internal nasal packing was done with merocele in 35 (38%) patients for 1 day. The average time taken for septoturbinoplasty was around 55 minutes. All the patients were admitted for 1 day and internal nasal packs were removed during discharge time. Routine follow up was done on 7th, 15th, 30th, and 60th day postoperatively to find out improvement in nasal obstruction using the NOSE scale.

Data was collected postoperatively and was analyzed using Statistical Packages for the Social Sciences version 16. Point estimate at 95% Confidence Interval was calculated, with frequency, percentage, mean and standard deviations.

## RESULTS

Out of 1248 patients, about 92 (7.37%) patients (4.95-10.14 at 95% Confidence Interval) underwent septoturbinoplasty using two different techniques. Regarding Turbinoplasty, 46 (50%) patients were operated by using Microdebrider Assisted Turbinoplasty (MAT) technique and the rest of the 46 (50%) by Out-fracture with Submucosal Diathermy (SMD) technique. Among them, 51 (55.4%) were male and 41 (44.6%) were female with age ranging from 15-50 years were enrolled in our study. Internal nasal packing was done with merocele in 35 (38%) patients for 1 day.

**Figure 1:**
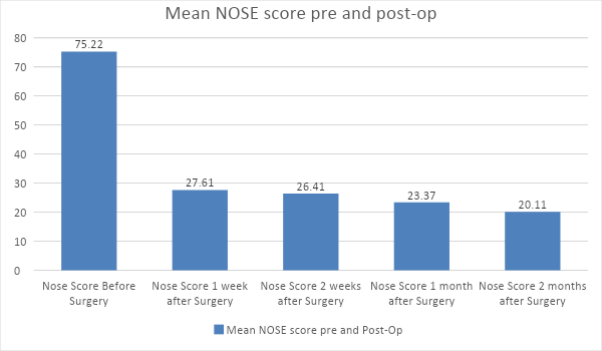
NOSE score before and after surgery (1 week, 2 weeks, 1 month, 2 months).

The mean NOSE score before surgery was 75.21±6.19. Likewise, the mean NOSE score after 1 week of surgery was 27.60±4.41, after 2 weeks was 26.41 ±3.34, after 1 month was 23.36 ±3.06 and after 2 months was 20.10±3.14 (Table 1, Figure 1).

**Table 1:** Mean NOSE score before and after surgery.

NOSE Score	Mean±SD	Minimum	Maximum
Before surgery	75.22±6.19	60	90
1 week after surgery	27.61±4.41	20	40
2 weeks after surgery	26.41±3.34	20	35
1 month after surgery	23.37±3.07	15	30
2 months after surgery	20.11±3.14	10	25

The mean NOSE score before and after surgery of both the techniques are shown in table 2 (Table 2).

**Table 2:** Mean NOSE score before and after surgery.

NOSE Score	Mean±SD
**Microdebrider Assisted Turbinoplasty**	
Before surgery	77.17±5.54
1 week after surgery	26.74±4.11
2 weeks after surgery	27.61±3.24
1 month after surgery	23.69±3.24
2 months after surgery	18.48±2.76
**Submucosal Diathermy**	
Before surgery	73.26±6.25
1 week after surgery	28.48±4.58
2 weeks after surgery	25.22±2.97
1 month after surgery	23.04±2.88
2 months after surgery	21.74±2.63

## DISCUSSION

Nasal obstruction is one of the commonest symptoms that patient presents in an ENT OPD.^12^ A large variety of diseases can cause different degree of nasal blockage, of which Inferior Turbinate Hypertrophy is one of the common causes.^13^ Deviated nasal septum and ITH have been the most common causes of nasal obstruction. Patients undergo Septoplasty as a management of deviated nasal septum yet they complain of nasal obstruction which is mostly due to unaddressed ITH. Most of the cases respond to antihistamines or local decongestant, however; surgery is needed if the ITH is due to submucous fibrosis rendering the turbinate incapable of decongestion.^10^ Septoturbinoplasty has an additive effect for the improvement of nasal obstruction. The basic principle of turbinoplasty is to reduce the turbinate size but the techniques differ from each other based on preservation of normal physiological function.

In our study we found that the mean NOSE score before surgery was 75.2 ±6.19. Likewise, the mean NOSE score after 1 week of surgery was 27.60±4.41, after 2 weeks was 26.41 ±3.34, after 1 month was 23.36±3.06 and after 2 months was 20.10±3.14. Postoperatively all the patients reported improved symptom scores of nasal obstruction, nasal stuffiness, snoring, oral breathing and better health related quality of life (HQOL) which is similar to the study done by Nilsen, et al.^14^ In both the MAT and SMD groups, similar results on 7th, 15th and 30th postoperative day was found, however on 60th postoperative day better results were seen in MAT group than out-fracture and SMD group. Similar results were seen in the study done by Joniau, et al.^15^ found that powered turbinoplasty was superior to submucosal cauterization in all aspects of the assessment.

Microdebrider Assisted Turbinoplasty (MAT) is relatively a new method for reducing the size of inferior turbinate. The complications associated with standard submucosal resection such as excessive resection of ITH, bleeding and crusting were largely avoided by MAT. In addition, this technique of turbinate reduction has been shown to be a reliable and safe method.^16–18^ Many authors used the microdebrider intraturbinally, few others used it extraturbinally for turbinate size reduction. We used a microdebrider intraturbinally to preserve the normal physiological function of mucosa. MAT is more difficult and has a higher complication rate than the out-fracture method, despite its high success rate. Inferior turbinate out-fracture and Submucosal Diathermy (SMD) is a safer and quickier technique,^19^ which we have used in 50% of our patients.

Our study showed 94% of the patients had complete resolution of nasal obstruction and stuffiness, while 6% of the patients had mild obstruction in 2 months follow-up in the MAT group. Similar study done by Friedman, et al.^18^ had found that symptoms of bilateral nasal obstruction and stuffiness were almost completely resolved after 6 weeks of MAT. Complete resolution of nasal blockage in 80% of patients and mild nasal obstruction in 20% of the patients two months after MAT was seen in the study done by Hegazy, et al.^20^ Mahlon, et al. performed MAT for 100 patients in the period from 1994 to 1997 and found that postoperative improvement in nasal patency occurred in 93% of the patients.^21^

We found 90% of the patients had complete resolution of nasal obstruction and stuffiness, while 10% of the patients had mild obstruction in 2 months follow-up in the Out-fracture and SMD group. Similar study done by Ehab, et al.^22^ found relief of nasal obstruction in 75% of the patients after one month of SMD and improvement of nasal patency was seen in all patients after three months. Fradis, et al.^23^ found that diathermy showed good results in 78% of cases two weeks after surgery. The efficacy of the procedure was reduced to 76% two months after surgery. Milo, et al.^24^ had found 70.3% experienced subjective improvement in nasal breathing after 2 months of SMD. Our results regarding improvement in nasal patency differ from the results obtained by other studies, these differences present because of out-fracture of inferior turbinate along with SMD in our study unlike only SMD in other studies.

The mean operative time for MAT was 30 minutes whereas that for SMD was 14 minutes; which is similar to the study done by Chen, et al.^25^ Diaa El Din, et al.^26^ found that intraoperative blood loss was (37.1±7.4)ml during MAT. We found (50.2±4.2)ml of intraoperative blood loss in both the techniques. Mucosal tears in MAT group were 13% and SMD group were 8% in our study which is similar to the study done by Liu, et al.^27^ noted (11.7%) mucosal tears in the microdebrider group. We found 38% of our patients had postoperative crusting on 7th day follow-up and was reduced to 2% on 2 months follow-up. Ehab, et al.^22^ found that crustation after SMD develops in 50% of patients in the first week, and this was reduced to 5% after 3 months. We found postoperative synaechiae in (3.26%) of our patients. Similar study done by Friedman, et al.^18^ observed postoperative synaechiae in 5% of the patients while Liu, et al.^27^ observed postoperative crusting and synaechiae in 11.66% of the patients.

Since, the study is a descriptive cross-sectional study and done in single hospital, the findings of the study cannot be generalized to whole population.

## CONCLUSIONS

The prevalence of septoturbinoplasty in our study is similar to other studies done in similar settings. We found almost complete resolution of breathing problems following endoscopic septoturbinoplasty, hence improving quality of life. Both the techniques are mucosal preservation techniques, but the results of MAT are more predictable in long term follow-up. Endoscopic guidance provided excellent visualization and intra-operative evaluation regarding the amount of turbinate tissue needed to be removed to achieve good patency and avoid complications.
